# Airway *Corynebacterium* interfere with *Streptococcus pneumoniae* and *Staphylococcus aureus* infection and express secreted factors selectively targeting each pathogen

**DOI:** 10.1128/iai.00445-24

**Published:** 2024-12-20

**Authors:** Emily Tamkin, Brian P. Lorenz, Arianna McCarty, Sam Fulte, Elan Eisenmesser, Alexander R. Horswill, Sarah E. Clark

**Affiliations:** 1 Department of Otolaryngology, University of Colorado School of Medicine 12225 , Aurora, Colorado, USA; 2 Department of Biochemistry and Molecular Genetics, University of Colorado School of Medicine 12225 , Aurora, Colorado, USA; 3 Department of Immunology and Microbiology, University of Colorado School of Medicine 12225 , Aurora, Colorado, USA; Georgia Institute of Technology, Atlanta, Georgia, USA

**Keywords:** *Streptococcus pneumoniae*, *Staphylococcus aureus*, *Corynebacterium*, respiratory tract infection, epithelial cells, hemolysins, adherence, microbial interactions

## Abstract

The composition of the respiratory tract microbiome is a notable predictor of infection-related morbidities and mortalities among both adults and children. Species of *Corynebacterium,* which are largely present as commensals in the upper airway and other body sites, are associated with lower colonization rates of opportunistic bacterial pathogens such as *Streptococcus pneumoniae* and *Staphylococcus aureus*. In this study, *Corynebacterium*-mediated protective effects against *S. pneumoniae* and *S. aureus* were directly compared using *in vivo* and *in vitro* models. Pre-exposure to *Corynebacterium pseudodiphtheriticum* reduced the ability of *S. aureus* and *S. pneumoniae* to infect the lungs of mice, indicating a broadly protective effect. Adherence of both pathogens to human respiratory tract epithelial cells was significantly impaired following pre-exposure to C. *pseudodiphtheriticum* or *Corynebacterium accolens*, and this effect was dependent on live *Corynebacterium* colonizing the epithelial cells. However, *Corynebacterium*-secreted factors had distinct effects on each pathogen. *Corynebacterium* lipase activity was bactericidal against *S. pneumoniae*, but not *S. aureus*. Instead, the hemolytic activity of pore-forming toxins produced by *S. aureus* was directly blocked by a novel *Corynebacterium*-secreted factor with protease activity. Taken together, these results suggest diverse mechanisms by which *Corynebacterium* contribute to the protective effect of the airway microbiome against opportunistic bacterial pathogens.

## INTRODUCTION

*Streptococcus pneumoniae* and *Staphylococcus aureus* are the predominant bacterial pathogens of the respiratory tract. *S. pneumoniae* (the pneumococcus) is a Gram-positive facultative anaerobe associated with a wide range of infections including pneumonia, bacteremia, otitis media, meningitis, and sinusitis (1). It is estimated that pneumonia accounts for 22% of all deaths in children aged 1 to 5 years (2) with *S. pneumoniae* identified as the leading cause of community-acquired pneumonia (3). *S. pneumoniae* colonizes the nasopharynx at variable rates among different populations, ranging from 5% to 70% worldwide, often functioning as a commensal with no physiological presentation (4). However, colonization in the upper airway is an important precursor of lower airway infection. *S. aureus* is another Gram-positive, facultative anaerobic bacterium that causes a wide range of clinical diseases. *S. aureus* asymptomatically colonizes the anterior nares of 20%–30% of the population (5), with carriers at increased risk for distal infections including skin and soft tissue infections, endocarditis, osteomyelitis, urinary tract infections, device-associated infections, and bacterial pneumonia. *S. pneumoniae* and *S. aureus* are also leading causes of secondary bacterial infection, which is associated with poorer clinical outcomes in patients with viral pneumonia (6, 7).

While antibiotics have proven effective since their widespread introduction in the 1940s, antibiotic resistance among pneumococcal and *S. aureus* infections has been rising in recent decades. Resistance to one or more antibiotics has been identified for around 30% of *S. pneumoniae* infections (8). Methicillin-resistant *S. aureus* (MRSA) is one of the leading causes of hospital-acquired infections and is associated with higher mortality due to treatment difficulty (9). There is currently no vaccine for *S. aureus*, limiting prevention to basic strategies such as hygiene and contact prevention along with anti-infectives (10). While the most recent iterations of pneumococcal vaccines target up to 23 serotypes of *S. pneumoniae*, the vast majority of the over 100 serotypes in circulation remain outside vaccine coverage. These difficulties underscore the need for new treatment and prevention strategies to limit the clinical burden of bacterial pneumonia.

The airway microbiome inhabiting the upper respiratory tract is one of the first lines of defense against pathogens including *S. pneumoniae* and *S. aureus*. Recently, *Corynebacterium* species have emerged as an important component of the beneficial impact of the airway microbiome against colonization and infection with respiratory tract pathogens (11). The presence of *Corynebacterium* in the upper airway is correlated with reduced *Staphylococcus aureus* and *Streptococcus pneumoniae* colonization and the promotion of a more stable airway microbiome (12–16). Longitudinal studies in young children have revealed that a greater abundance of *Corynebacterium* is predictive of lower infection risk (17–20). Inoculation of *Corynebacterium* into the nares of human volunteers resulted in a significant reduction in *S. aureus* colonization (21), demonstrating as a proof-of-principle that *Corynebacterium* species can interfere with *S. aureus* colonization. The species *Corynebacterium pseudodiphtheriticum* and *Corynebacterium accolens* have protective phenotypes in several models. In mice, pre-exposure to *C. pseudodiphtheriticum* reduced *S. pneumoniae* lung burdens in a viral co-infection model (22), and pre-exposure to *C. accolens* reduced *S. pneumoniae* colonization and lower airway infection (23). However, there is also evidence for species-specific effects of *Corynebacterium* isolates. In one study, *C. pseudodiphtheriticum* was negatively correlated with *S. aureus* carriage and reduced *S. aureus* growth on agar plates, while *C. accolens* had the opposite effect (15). In contrast, several nasal *C. accolens* isolates impaired *S. aureus* growth on agar plates and reduced *S. aureus* virulence in a worm infection model (24, 25). While these studies corroborate the inhibitory potential of *Corynebacterium* against *S. pneumoniae* and *S. aureus,* the mechanisms mediating *Corynebacterium* protective effects are still largely unknown and the species-specific effects on *S. pneumoniae* versus *S. aureus* remain unclear.

Here, we used a mouse infection model and several *in vitro* systems to interrogate the effects of *Corynebacterium* species against *S. pneumoniae* and *S. aureus* in parallel. Our findings identify *Corynebacterium* isolates with broad inhibitory effects against both pathogens in the context of lung infection and adherence to human respiratory tract epithelial cells, with distinct secreted factors contributing to pathogen-specific interference.

## MATERIALS AND METHODS

### Bacterial strains

All bacterial strains used in this study are listed in Table 1. *Corynebacterium* isolates were grown from glycerol stocks on BHI agar plates (BD Difco Bacto Brain Heart Infusion, Thermo Fisher Scientific) supplemented with 1% Tween 80 (polysorbate, VWR) aerobically at 37°C overnight. From agar plates, colonies were used to inoculate BHI + 1% Tween 80 liquid cultures which were grown aerobically for 18 hours at 37°C with shaking at 200 rpm. When indicated, 180 mg/mL of triolein was added to agar plates or as specified to liquid media cultures. Heat-killed (HK) *Corynebacterium* was prepared by resuspending pelleted bacteria from liquid cultures in phosphate buffered saline (PBS), washing three times in PBS, and incubating for 30 minutes at 65°C. Bacterial killing was confirmed by the absence of growth on agar plates.

**TABLE 1:** Bacterial strains^*c*^

Strain	Characteristics	Reference
*S. pneumoniae*		
*S. pneumoniae* D39	*rspL* streptomycin resistant	(26)
*S. pneumoniae* R6	Unencapsulated	(27)
*S. aureus*		
*S. aureus* USA300 (AH1263)	MRSA^*a*^	(28)
*S. aureus^ΔfnbAB^* (AH4392)	*fnbAB* mutant of USA300	(29)
*S. aureus^Δagr^* (AH1292)	*agr* mutant of USA300	(30)
*S. aureus^Δhla^* (AH1589)	*hla* mutant of USA300	(31)
*Corynebacterium*		
*C. pseudodiphtheriticum* DSM 44287		Leibniz Institute, DSMZ
*C. accolens* ATCC 49726		ATCC^*b*^
*C. accolens* KPL1818	Human nasal isolate	(32)
*C. accolens* KPL1818*^Δlips1^* (KPL2503)	*lips1* mutant of KPL1818	(32)
*C. accolens* KPL1818*^compl^* (KPL2505)	Complemented *lips1* mutant	(32)
*C. accolens* DSM 44278		Leibniz Institute, DSMZ
*C. accolens* DSM 44279		Leibniz Institute, DSMZ
*C. striatum* DSM 20668		Leibniz Institute, DSMZ
*C. amycolatum* SK46 BEI HM-109		BEI Resources, NIAID, NIH

^*a*^ MRSA, methicillin-resistant *S. aureus*.

^*b*^ ATCC, American Type Culture Collection.

^*c*^ Shaded rows denote each species or genus used in the study.

*S. pneumoniae* strains were cultured from frozen glycerol stocks in Todd Hewitt Broth with 5% Yeast Extract (BD Bacto) at 37°C with 5% CO_2_ to mid-log phase. For agar plates, tryptic soy broth (TSB, MP Biomedicals) was supplemented with 5 µg/mL of neomycin and 5,000 units/plate of fresh catalase (Worthington Biomedical Corporation), and plates were incubated at 37°C with 5% CO_2_ for 18 hours. Media was supplemented with 50 µg/mL streptomycin (MilliporeSigma) for the streptomycin-resistant variant of strain D39. *S. aureus* strains were cultured from frozen glycerol stocks on mannitol salt agar (Thermo Scientific Oxoid) for 18 hours at 37°C. Bacteria from agar plates were used to inoculate BHI liquid media and cultures were grown at 37°C with shaking at 200 rpm to mid-log phase.

### Mouse infection

Male and female C57BL/6J wild-type (WT) mice aged 6–12 weeks old were purchased from The Jackson Laboratory (RRID:IMSR_JAX:000664). Prior to infection, mice were treated with an antibiotic cocktail (ampicillin 1 g/L, neomycin 1 g/L, metronidazole 1 g/L, and vancomycin 0.5 g/L, MilliporeSigma and McKesson) *ad libitum* for 14 days, with a 48-hour rest on regular water prior to *C. pseudodiphtheriticum* exposures. All infections were performed intranasally under inhaled isoflurane anesthesia with doses of 10^7^–10^8^ colony forming units (CFUs)/mouse, as indicated. *C. pseudodiphtheriticum* was prepared for infection as pellets re-suspended in 25 µL of cell-free conditioned media (CFCM), described below. Mice were exposed to two consecutive doses of *C. pseudodiphtheriticum* at 24-hour intervals, with the final exposure occurring 24 hours prior to pathogen infection. *S. aureus* and *S. pneumoniae* were prepared as pellets re-suspended in 25 µL of PBS. Mice were sacrificed 24 hours post-infection (hpi) with *S. aureus* or *S. pneumoniae* and nasal lavages and bronchoalveolar lavages, and lungs were collected. Nasal lavages were performed by instillation of 200 µL PBS with a cannulated trachea through the nasal cavity and collected from the nares. Bronchoalveolar lavage was collected through cannulated tracheas in 1 mL 1× PBS. Lungs were homogenized in 1× PBS using a Bullet Blender tissue homogenizer (Stellar Scientific, Baltimore, MD). Lung homogenates were centrifuged for 30 seconds at 500 × *g* prior to serial dilution and plating on selective agar media. CFU burdens in the lungs are reported as a combined measure of bronchoalveolar lavage and lung tissue homogenate burdens.

### CFCM plate assays

*Corynebacterium* were grown on BHI + 1% Tween 80 agar plates supplemented with or without triolein spread on plates as a 180 mg/mL emulsion in 70% ethanol. Inoculated plates were incubated 18 hours under aerobic conditions at 37°C. Liquid cultures prepared from plates were grown in BHI + 1% Tween 80 broth with and without 45 mg/mL triolein and grown 18 hours at 37°C with shaking at 200 rpm. CFCM was prepared from supernatants of overnight liquid cultures following centrifugation at ≥20,000 × *g* for 10 minutes and 0.22 µm filtration. CFCM was spread as 200 µL/plate.

### Epithelial cell adherence assays

The human lung epithelial cell line A549 and pharyngeal epithelial cell line D562 were obtained from the American Type Culture Collection. Epithelial cells were cultured in Ham’s F-12K (Life Technologies Corporation) supplemented with 10% fetal bovine serum (FBS; CPS serum) and 1× penicillin-streptomycin (Life Technologies Corp.) at 37°C with 5% CO_2_. For adherence assays, 2 × 10^5^ cells/well were seeded into 24-well culture plates 48 hours prior to infection in Ham’s F-12K media with 10% FBS without antibiotics. At 48 hours, cells were washed once with 1× PBS, twice with HBSS(-) (Life Technologies Corp.), and cultured in Ham’s F-12K media with 10% FBS without antibiotics. Multiplicity of infection (MOI) was determined based on epithelial cell counts obtained following treatment with trypsin-EDTA (Sigma-Aldrich) for 5–6 minutes at 37°C with 5% CO_2_ and quantification on a hemocytometer following the addition of Trypan Blue to exclude dead cells (Acros Organics). After addition of *Corynebacterium* at the indicated MOI, plates were centrifuged for 3 minutes at 1,000 × *g* to support bacterial adherence and plates were incubated 18 h at 37°C with 5% CO_2_. At 18 hours, *S. pneumoniae* strain R6 or *S. aureus* was added as a PBS cell suspension at the indicated MOI and plates were centrifuged for 3 minutes at 1,000 × *g*. Following 1-hour infection at 37°C with 5% CO_2_, media was aspirated and cells were washed 3× with HBSS(-) and 1× with PBS. Cells were lysed following trypsin-EDTA digestion for 15 minutes at 37°C with 5% CO_2_, after which 300 µL of Milli-Q water was added to each well and mixed thoroughly to suspend bacteria. CFUs were enumerated from cell suspensions following serial dilution and plating on selective agar media. Percent adherence was calculated relative to CFUs obtained from media controls consisting of wells without epithelial cells. Normalized percent adherence was calculated relative to pathogen adherence in untreated wells not colonized with *Corynebacterium*. Percent cytotoxicity was evaluated by measurement of lactate dehydrogenase release in culture supernatants using a Cytotoxicity Assay Kit (Cayman Chemical).

### Hemolysis assays

For the zone of clearance (ZOC) hemolysis assay, 200 µL of CFCM was spread onto Columbia Blood Agar plates (BBL Columbia CNA Agar w/ 5% sheep blood, BD) and allowed to dry completely. Serial dilutions of *S. aureus* liquid cultures were spread onto plates in 20 µL spots to facilitate quantification of individual colony zones of hemolysis. Photos taken at 18 hours with a reference ruler were used to calculate ZOC in Adobe Photoshop CC, 2024. For the human blood hemolysis assay, whole blood collected from healthy adults was used to purify red blood cells (RBCs) by centrifugation at 1,000 × *g* for 10 minutes. CFCM was prepared from *S. aureus* and *Corynebacterium* liquid cultures, as described above. Reaction mixtures consisted of 75 µL of CFCM, 25 µL TSB, 100 µL 2× HA buffer (8.5 mL 10% NaCl, 2 mL 1M CaCl_2_, and 39.5 mL sterile water), and 25 µL RBCs diluted 1:5 in PBS. Tubes were gently inverted to mix and placed on a tube rotator a 37°C. At each experimental time point, tubes were removed and spun at 5,000 × *g* for 1 minute to pellet intact RBCs. Supernatants were used to measure absorbance at OD_543_. Heat-treated *Corynebacterium* CFCM was prepared by incubation at 100°C for 30 minutes. For protease inhibition, 50 µL protease inhibitor (Halt Protease Inhibitor Single-Use Cocktail, Thermo Fisher Scientific) was added to CFCM. Control CFCM was treated with equivalent volume of dimethylsulfoxide, DMSO (Thermo Fisher Scientific).

### Statistical analysis

GraphPad Prism (version 10, GraphPad Software LLC, San Diego, CA) was used to complete graphing and statistical analyses. Statistical tests included the Student’s *t*-test and analysis of variance (ANOVA) as appropriate based on the number of group comparisons. Mann-Whitney U-tests and Kruskal-Wallis tests were used for CFU data, which had non-normal (Gaussian) distribution due to the limit of detection cut-off. For all tests, *P* < 0.05 was considered significant.

## RESULTS

### *C. pseudodiphtheriticum* protects against airway pathogen infection

**Fig 1:**
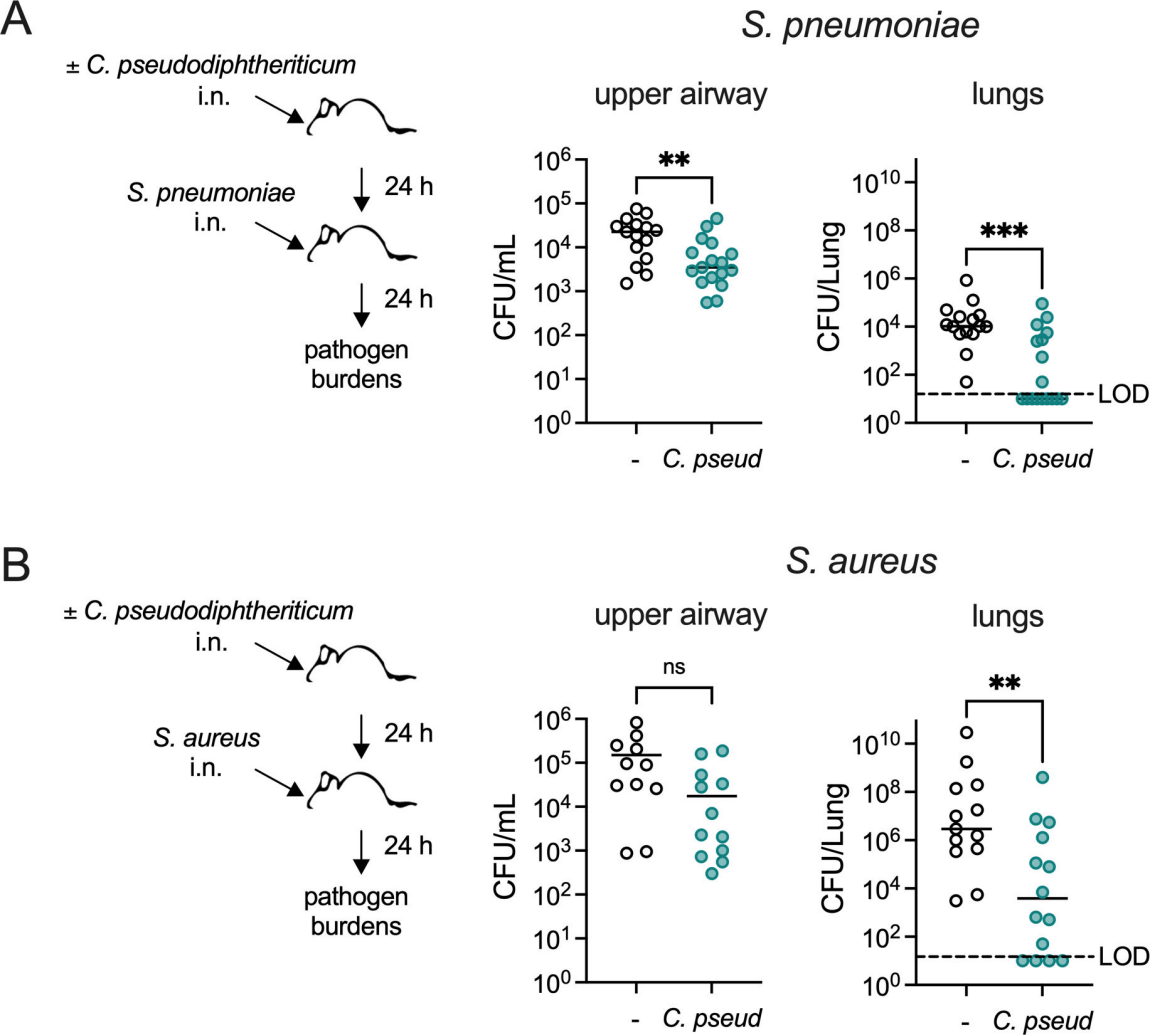
Exposure to *C. pseudodiphtheriticum* reduces *S. pneumoniae* and *S. aureus* respiratory tract infection. (**A**) Burdens of *S. pneumoniae* type 2 strain D39 detected in mice at 24 hours post-infection with 10^7^ CFU/mouse i.n., with or without pre-exposure to 10^7^ CFU/mouse *C*. *pseudodiphtheriticum* (*C. pseud*) i.n. Mice were treated with antibiotics for 2 weeks prior to bacterial exposures. Upper airway CFUs were enumerated from nasopharyngeal lavage fluid. Lung CFUs were the total detected from bronchoalveolar lavage fluid and lung tissue. (**B**) Burdens of *S. aureus* MRSA strain USA300 detected in mice treated as in (**A**) at 24 hours post-infection with 10^8^ CFU/mouse i.n. **P < 0.01, ***P < 0.001, Mann-Whitney U-test. Data are pooled from three independent experiments with *n* = 15–17 mice/group (**A**) or *n* = 13–14 mice/group (**B**). LOD indicates the limit of detection for CFUs.

To directly compare the impact of *C. pseudodiphtheriticum* on susceptibility to infection with *S. pneumoniae* and *S. aureus*, we used a murine respiratory tract infection model developed in prior work (23). In accordance with this model, C57BL/6J wild-type mice were treated with antibiotics for 2 weeks to facilitate *Corynebacterium* colonization (23). Antibiotic-treated mice were intranasally inoculated with *C. pseudodiphtheriticum* 24 hours prior to intranasal infection with either encapsulated serotype 2 *S*. *pneumoniae* strain D39 or USA300 MRSA *S. aureus* strain LAC. *S. aureus* and *S. pneumoniae* burdens were quantified as CFUs in nasal lavage fluid, bronchoalveolar lavage fluid, and lung tissue homogenates collected 24 hours post-infection. *S. pneumoniae* burdens in the upper and lower airways were significantly reduced in mice pre-exposed to *C. pseudodiphtheriticum* (Fig. 1A), indicating that *C. pseudodiphtheriticum* provides a protective effect against *S. pneumoniae* nasopharyngeal colonization and lung infection. Similarly, *S. aureus* burdens were lower in mice pre-exposed to *C. pseudodiphtheriticum*, with a significant reduction in the lungs (Fig. 1B). Burdens in the lower airway at this early time point are a consequence of both bacterial aspiration to the lungs during intranasal infection and invasion to the lungs following upper airway colonization. In the lungs, 53% of the mice pre-exposed to *Corynebacterium* had no detectable infection with *S. pneumoniae*, compared to 0% of mice without *Corynebacterium* pre-exposure, indicating an improvement in either preventing the establishment of pneumococcal lung infection or in clearance of the infection. *Corynebacterium* also reduced acquisition of *S. aureus* lung infection, as 21% of mice pre-exposed to *C. pseudodiphtheriticum* had no detectable infection compared with 0% in the group without *Corynebacterium* pre-exposure. Taken together, these results suggest that *C. pseudodiphtheriticum* has a dual protective effect against respiratory tract infection with *S. pneumoniae* and *S. aureus*.

### *Corynebacterium*-secreted lipase selectively inhibits *S. pneumoniae*

**Fig 2:**
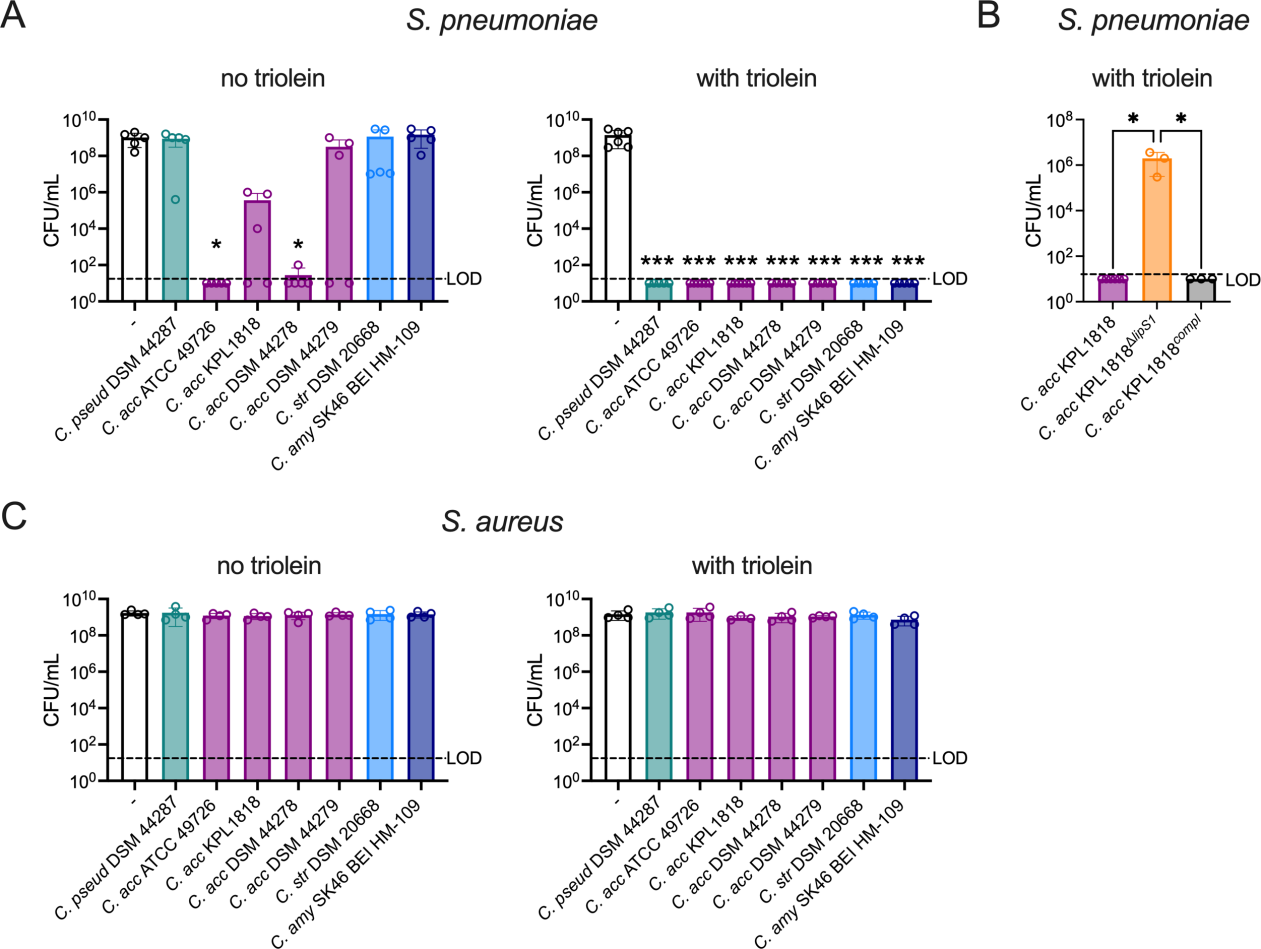
*Corynebacterium*-secreted lipase inhibits *S. pneumoniae* growth without affecting *S. aureus*. (**A**) Growth of *S. pneumoniae* type 2 strain D39 detected on tryptic soy broth agar following pre-spreading plates with supernatants from *C. pseudodiphtheriticum* (*C. pseud* DSM 44287), *C. accolens* (*C. acc* ATCC 49726, *C. acc* KPL1818, *C. acc* DSM 44278, *C. acc* DSM 44279), *C. striatum* (*C. str*), or *C. amycolatum* (*C. amy*) strains as indicated grown with 1% Tween 80 alone (no triolein) or supplemented with 180 mg/mL triolein (with triolein). (**B**) Growth of *S. pneumoniae* as for (**A**) following pre-spreading plates with supernatants from *C. accolens* WT (*C. acc* KPL1818), a *C. accolens* mutant deficient in *lipS1 (C. acc* KPL1818^Δ^*^lipS1^*), or a *lipS1* complemented strain (*C. acc* KPL1818*^compl^*) grown with 1% Tween 80 and supplemented with triolein. (**C**) Growth of *S. aureus* MRSA strain USA300 detected on mannitol salt agar as in (**A**). *P < 0.05, ***P < 0.001, Kruskal-Wallis test with Dunn’s *post hoc* analysis. Data are pooled from three to four independent experiments. LOD indicates the limit of detection.

Lipases and esterases secreted by *Corynebacterium* species mediate direct inhibition of *S. pneumoniae* growth through the production of bactericidal free fatty acids following cleavage of host lipids (23, 32). In *C. accolens*, the lipase LipS1 is required for *S. pneumoniae* inhibition in the presence of triolein, a naturally occurring triacylglycerol (32). LipS1 partially contributed to reduced *S. pneumoniae* infection following *C. accolens* pre-exposure in a mouse lung infection model (23). To better understand the relationship between the availability of exogenous lipids and pneumococcal inhibition, a range of *Corynebacterium* isolates was surveyed for their effect on *S. pneumoniae* growth using CFCM. When supplemental lipid was limited to the 1% Tween 80 in growth media, *Corynebacterium* isolates showed a range of pneumococcal growth inhibition (Fig. 2A). Among these, CFCM from two different *C. accolens* strains completely inhibited *S. pneumoniae* growth. In contrast, CFCM isolated from strains grown in media supplemented with triolein and 1% Tween 80 conferred complete inhibition of *S. pneumoniae* growth for all isolates tested, which included one *C. pseudodiphtheriticum* isolate, four *C. accolens* isolates, one isolate of *Corynebacterium striatum*, and one isolate of *Corynebacterium amycolatum* (Fig. 2A). *C. accolens* restriction of *S. pneumoniae* growth in the presence of triolein was dependent on LipS1, as previously reported (32), based on the recovery of *S. pneumoniae* growth following exposure to CFCM from LipS1-deficient *C. accolens^Δlips1^*, but not in CFCM from the complemented strain (Fig. 2B). As *Corynebacterium* clinical isolates are frequently tested for pathogen-inhibitory capacity, these data suggest that the inclusion of exogenous triolein is critical to capture the full spectrum of *S. pneumoniae* growth inhibition.

While *Corynebacterium* species have also been reported to reduce *S. aureus* growth on agar plates (15, 24, 33), it is unclear whether secreted lipase and/or esterase activity contributes to this phenotype. Using an identical experimental setup as for *S. pneumoniae*, we found that none of the *Corynebacterium* isolates tested inhibited *S. aureus* growth, regardless of triolein supplementation (Fig. 2C). These findings suggest that secreted lipid catabolizing enzymes in *Corynebacterium* species selectively inhibit growth of *S. pneumoniae*, but not *S. aureus*.

### *C. pseudodiphtheriticum* reduces pathogen adherence to human respiratory tract epithelial cells

**Fig 3:**
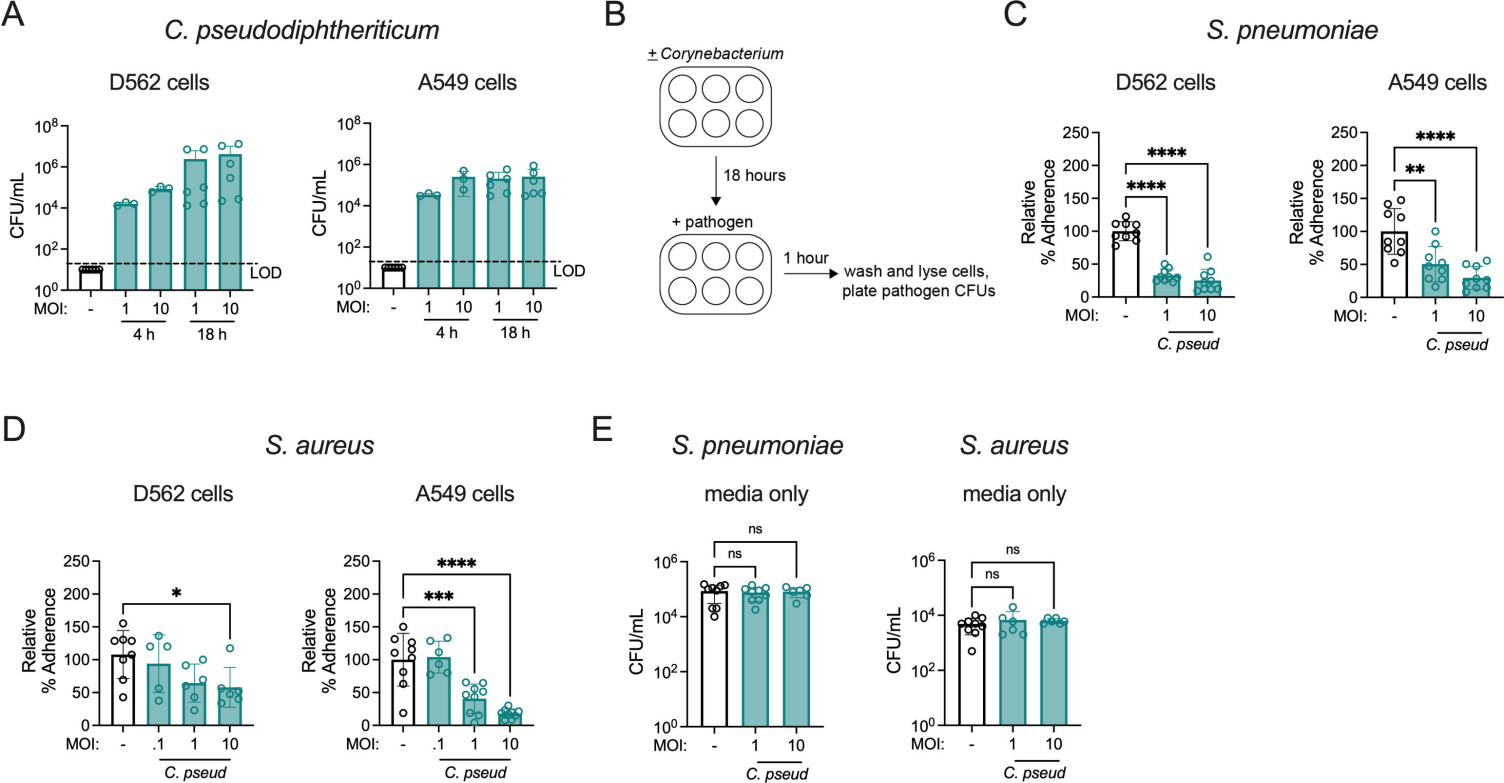
*C. pseudodiphtheriticum* colonization reduces adherence of *S. pneumoniae* and *S. aureus* to human respiratory tract epithelial cells. (**A**) Burdens of *C. pseudodiphtheriticum* detected on D562 and A549 cells at 4 hours and 18 hours at the indicated MOI. LOD indicates the limit of detection. (**B**) Cell adherence assay schematic. (**C**) Percent adherence of *S. pneumoniae* unencapsulated strain R6 detected on D526 and A549 cells at 1 hour post-infection at MOI = 0.1 with or without pre-colonization of epithelial cells with *C. pseudodiphtheriticum* for 18 hours at the indicated MOI, relative to untreated cells infected with *S. pneumoniae* alone. (**D**) Percent adherence of *S. aureus* MRSA strain USA300 at MOI = 0.1 as for (**C**). (**E**) Burdens of *S. pneumoniae* and *S. aureus* detected in wells without epithelial cells under identical conditions as for (**C and D**). *P < 0.05, **P < 0.01, ****P < 0.0001, one-way ANOVA with Dunnett’s *post hoc* analysis. Data are pooled from two independent experiments (**A**) or three independent experiments (**C–E**) with three replicates per condition.

Given the inhibitory effect of *C. pseudodiphtheriticum* against both *S. aureus* and *S. pneumoniae* infection *in vivo*, we next addressed whether *Corynebacterium* colonization has a similarly broad impact on pathogen adherence to human respiratory tract epithelial cells. The pharyngeal cell line D562 and lung alveolar cell line A549 are well-established models for investigation of *S. pneumoniae* and *S. aureus* adherence to the airway epithelium (34–37). *C. pseudodiphtheriticum* colonized both A549 and D562 cells, establishing burdens of ~10^4^ CFU/mL after 4 hours and 10^5-6^ CFU/mL by 18 hours post-inoculation (Fig. 3A), with no detectable cytotoxicity (Fig. S1A). Successful epithelial cell colonization with *C. pseudodiphtheriticum* enabled us to address the effect of pre-exposure on pathogen adherence, similar to the *in vivo* challenge model. Following an 18-hour colonization with *C. pseudodiphtheriticum*, D562 and A549 cells were infected with *S. aureus* or *S. pneumoniae* and adherence was calculated relative to control wells containing media without epithelial cells (Fig. 3B). Unencapsulated *S. pneumoniae* strain R6 was used to facilitate pneumococcal adherence to epithelial cells, which is lower in encapsulated strains (35). *C. pseudodiphtheriticum* colonization significantly reduced *S. pneumoniae* and *S. aureus* adherence to both D562 and A549 epithelial cells (Fig. 3C and D). The effect of *C. pseudodiphtheriticum* on pathogen adherence was dependent on the MOI, as *S. aureus* adherence was similar to non-colonized epithelial cells at lower *C. pseudodiphtheriticum* MOIs. However, *S. aureus* adherence was still significantly reduced by *C. pseudodiphtheriticum* colonization when the pathogen MOI was increased to 10, indicating successful interreference at a ratio as low as 1 *Corynebacterium* per 10 *S. aureus* bacterial cells (Fig. S1B). Identical conditions in wells without epithelial cells demonstrated that there was no direct pathogen killing effect under these conditions (Fig. 3E).

### *C. accolens* reduces pathogen adherence to epithelial cells in a lipase-independent manner

**Fig 4:**
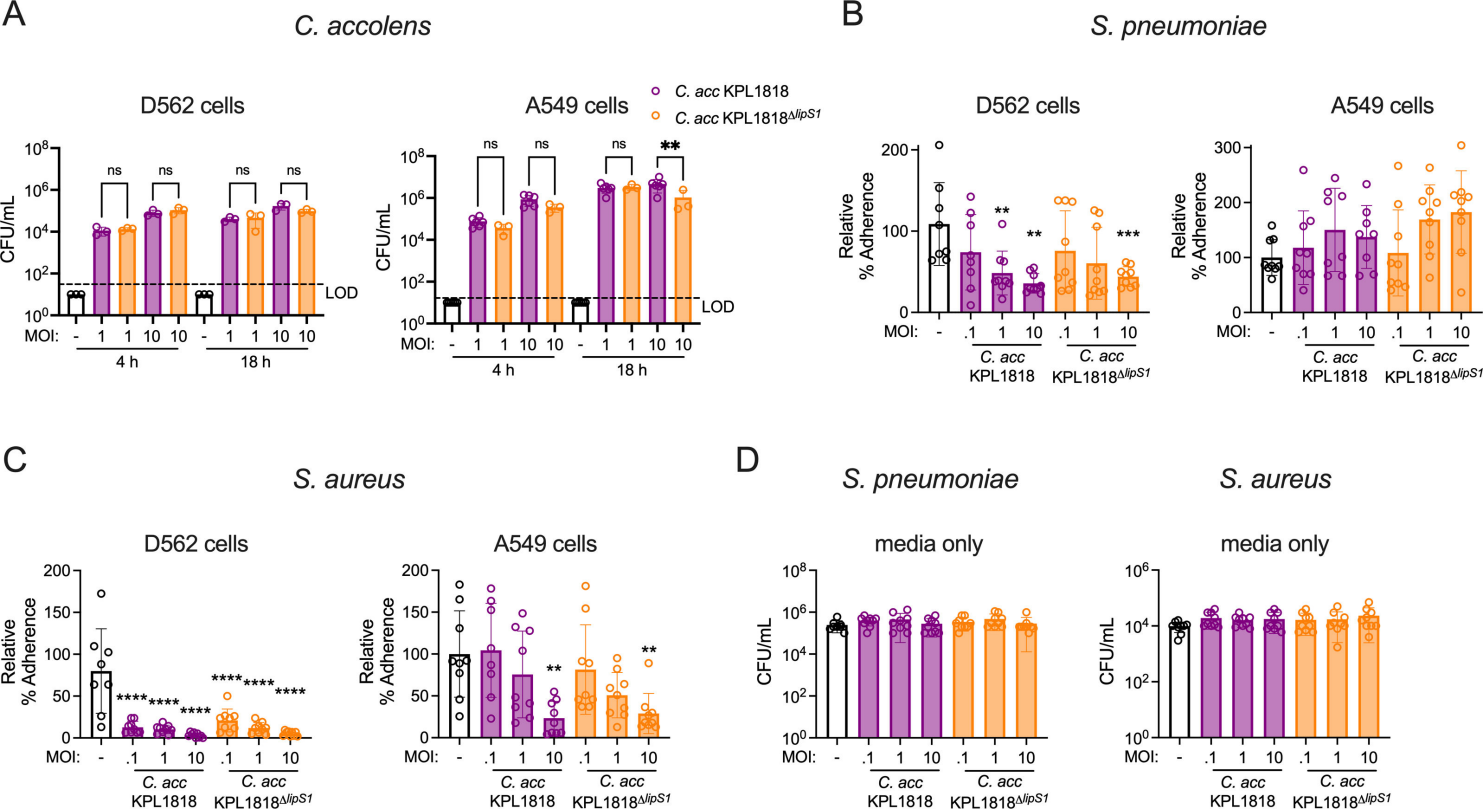
*C. accolens* colonization reduces pathogen adherence to human respiratory tract epithelial cells in a lipase-independent manner. (**A**) Burdens of *C. accolens* detected on D562 and A549 cells at 4 hours and 18 hours at the indicated MOI. LOD indicates the limit of detection. (**B**) Percent adherence of *S. pneumoniae* unencapsulated strain R6 detected on D526 and A549 cells at 1 hour post-infection at MOI = 0.1 with or without pre-colonization of epithelial cells with *C. accolens* WT (*C. acc* KPL1818) or *lipS1*-deficient *C. accolens* (*C. acc* KPL1818^Δ^*^lipS1^*) for 18 hours at the indicated MOI, relative to untreated cells infected with *S. pneumoniae* alone. (**C**) Percent adherence of *S. aureus* MRSA strain USA300 at MOI = 0.1 as for (**B**). (**D**) Burdens of *S. pneumoniae* and *S. aureus* detected in wells without epithelial cells under identical conditions as for (**B and C**). **P < 0.01, ***P < 0.001, ****P < 0.0001, one-way ANOVA with Dunnett’s *post hoc* analysis. Data are pooled from two independent experiments (**A**) or three independent experiments (**B–D**) with three replicates per condition.

To establish whether the colonization-associated protective effect against pathogen adherence extended to other *Corynebacterium* species, the same experimental set-up was used with *C. accolens*. First, *C. accolens* was confirmed to colonize D562 and A549 epithelial cells, with similar burdens established by 4 hours for both wild-type *C. accolens* and *C. accolens^Δlips1^* (Fig. 4A). Burdens were also largely similar regardless of LipS1 expression at 18 hours, apart from reduced colonization for the LipS1-deficient strain at the highest MOI tested in A549 cells. *C. accolens* colonization significantly reduced *S. pneumoniae* adherence to D562 cells, and this effect was dependent on the *Corynebacterium* MOI (Fig. 4B), as with *C. pseudodiphtheriticum*. For D562 cells, on which *C. accolens* wild-type and LipS1-deficient strains colonized equally well, *S. pneumoniae* adherence was reduced following colonization with either wild-type *C. accolens* or *C. accolens^Δlips1^*. In contrast, *C. accolens* colonization was not effective against *S. pneumoniae* adherence to A549 cells, which was similar regardless of *C. accolens* or *C. accolens^Δlips1^* pre-exposure (Fig. 4B), indicating a stronger effect against pneumococcal adherence to pharyngeal epithelial cells common to the upper airway.

*C. accolens* interference with *S. aureus* adherence was more pronounced, with limited *S. aureus* adherence to D562 cells apparent even at the lowest *C. accolens* MOI tested, 0.1 (Fig. 4C). Furthermore, *C. accolens* also reduced *S. aureus* adherence to A549 cells, though this effect was only significant at the highest MOI, 10. The magnitude of *Corynebacterium* interference with pathogen adherence was strain-dependent, as another *C. accolens* isolate (ATCC strain 4926) was more effective at reducing *S. aureus* adherence to A549 cells, which was significantly lower at all *C. accolens* ATCC 4926 MOIs tested, with the greatest reduction at the highest MOI (Fig. S1C). Importantly, *C. accolens*-mediated impairment of *S. aureus* adherence was independent of LipS1 expression, as indicated by similar effects of *C. accolens* and *C. accolens^Δlips1^*. As with *C. pseudodiphtheriticum*, we observed no direct effect on pathogen growth under these conditions, based on similar *S. pneumoniae* and *S. aureus* burdens in control wells containing media without host cells (Fig. 4D). Together, these data suggest that *Corynebacterium* colonization of the respiratory tract epithelium is sufficient to reduce adherence of both *S. pneumoniae* and *S. aureus*, and this effect is largely independent of secreted lipase activity.

### Colonization interference requires live *Corynebacterium*

**Fig 5:**
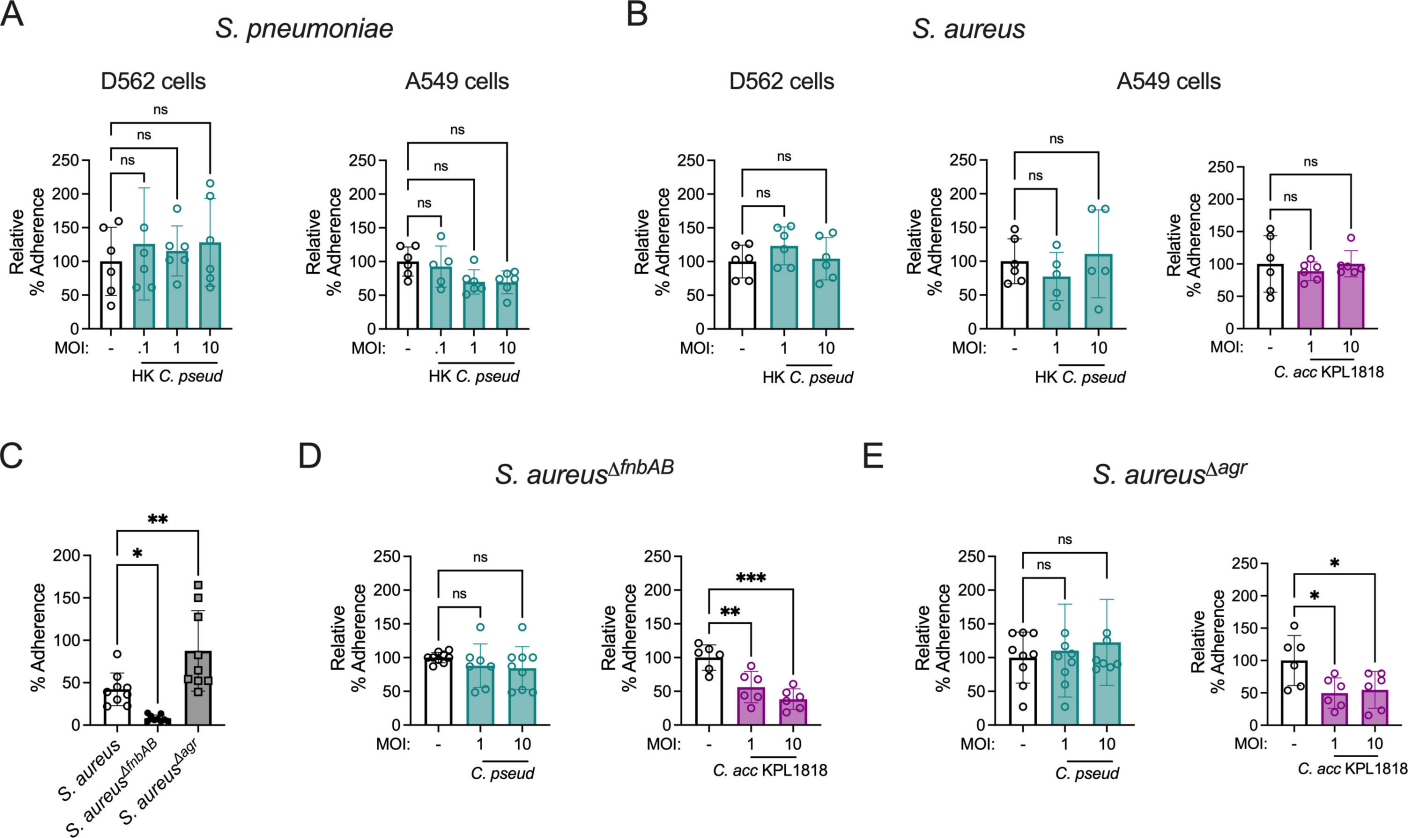
*Corynebacterium* colonization interference requires live bacteria and is sensitive to *S. aureus* adherence capacity. (**A**) Percent adherence of *S. pneumoniae* unencapsulated strain R6 on D562 and A549 cells 1 hour post-infection at MOI = 0.1 with or without 18 hour pre-exposure to heat-killed *C. pseudodiphtheriticum* (HK *C. pseud*) at the indicated MOI, relative to untreated cells infected with *S. pneumoniae* alone. (**B**) Percent adherence of *S. aureus* MRSA strain USA300 at MOI = 0.1 as for (**A**), with or without heat-killed *C. pseudodiphtheriticum* or heat-killed *C. accolens* WT (*C. acc* KPL1818). (**C**) Percent adherence of WT *S. aureus*, *fnbAB*-deficient *S. aureus* (*S. aureus*^Δ^*^fnbAB^*), and *agr*-deficient *S. aureus* (*S. aureus*^Δ^*^agr^*) to A549 cells 1 hour post-infection at MOI = 0.1. (**D**) Percent adherence of *S. aureus*^Δ^*^fnbAB^* to A549 cells 1 hour post-infection at MOI = 0.1 with or without pre-colonization of epithelial cells with *C. pseudodiphtheriticum* or *C. accolens* for 18 hours at the indicated MOI, relative to untreated cells infected with *S. aureus*^Δ^*^fnbAB^* alone. (**E**) Percent adherence of *S. aureus*^Δ^*^agr^* at MOI = 0.1 as in (**D**). *P < 0.05, **P < 0.01, one-way ANOVA with Dunnett’s *post hoc* analysis. Data are pooled from two (**A and B**) or three (**C–E**) independent experiments with three replicates per condition.

To define the requirements for *Corynebacterium* inhibition of pathogen adherence, we examined the impact of HK *C. pseudodiphtheriticum* on adherence of *S. pneumoniae* and *S. aureus* to A549 and D562 epithelial cells. HK *Corynebacterium* cannot establish a resident population on the epithelial cell surface but may retain the capacity to stimulate other cellular responses. However, HK *C. pseudodiphtheriticum* had no effect on *S. pneumoniae* or *S. aureus* adherence (Fig. 5A and B). Similarly, HK *C. accolens* had no effect on *S. aureus* adherence to A549 cells (Fig. 5B). These results suggest that live *Corynebacterium* are required for reduced pathogen adherence.

*S. aureus* adherence to epithelial cells relies on a family of fibronectin-binding proteins whose expression is suppressed by the accessory gene regulator (*agr*) system (29, 38–40). *S. aureus^ΔfnbAB^*, a fibronectin-binding protein knockout strain, was previously shown to have drastically reduced adherence to vaginal epithelial cells (41). Similarly, we found that *S. aureus^ΔfnbAB^* adherence to A549 lung epithelial cells was significantly lower than that of wild-type *S. aureus* (Fig. 5C). In contrast, an *agr*-deficient strain, *S. aureus^Δagr^*, was more adherent that wild-type *S. aureus*. Using relative adherence to compare the effect of *C. pseudodiphtheriticum* colonization on the low-adherent *S. aureus^ΔfnbAB^* mutant, we found that *C. pseudodiphtheriticum* was unable to reduce adherence any further (Fig. 5D). *C. pseudodiphtheriticum* colonization also had no impact on the highly adherent *S. aureus^Δagr^* mutant (Fig. 5E). Unlike *C. pseudodiphtheriticum*, *C. accolens* retained the ability to reduce both the low-adherent *aureus^ΔfnbAB^* mutant and the highly adherent *S. aureus^Δagr^* mutant (Fig. 5D and E). Together, these findings suggest that *C. pseudodiphtheriticum* impairment of *S. aureus* adherence is related to competition for space or binding sites on the epithelial surface between bacteria, rather than through an indirect cell stimulatory effect, whereas *C. accolens* blockade of *S. aureus* adherence may involve additional factors.

### *Corynebacterium*-secreted factor inhibits *S. aureus* hemolysis

**Fig 6:**
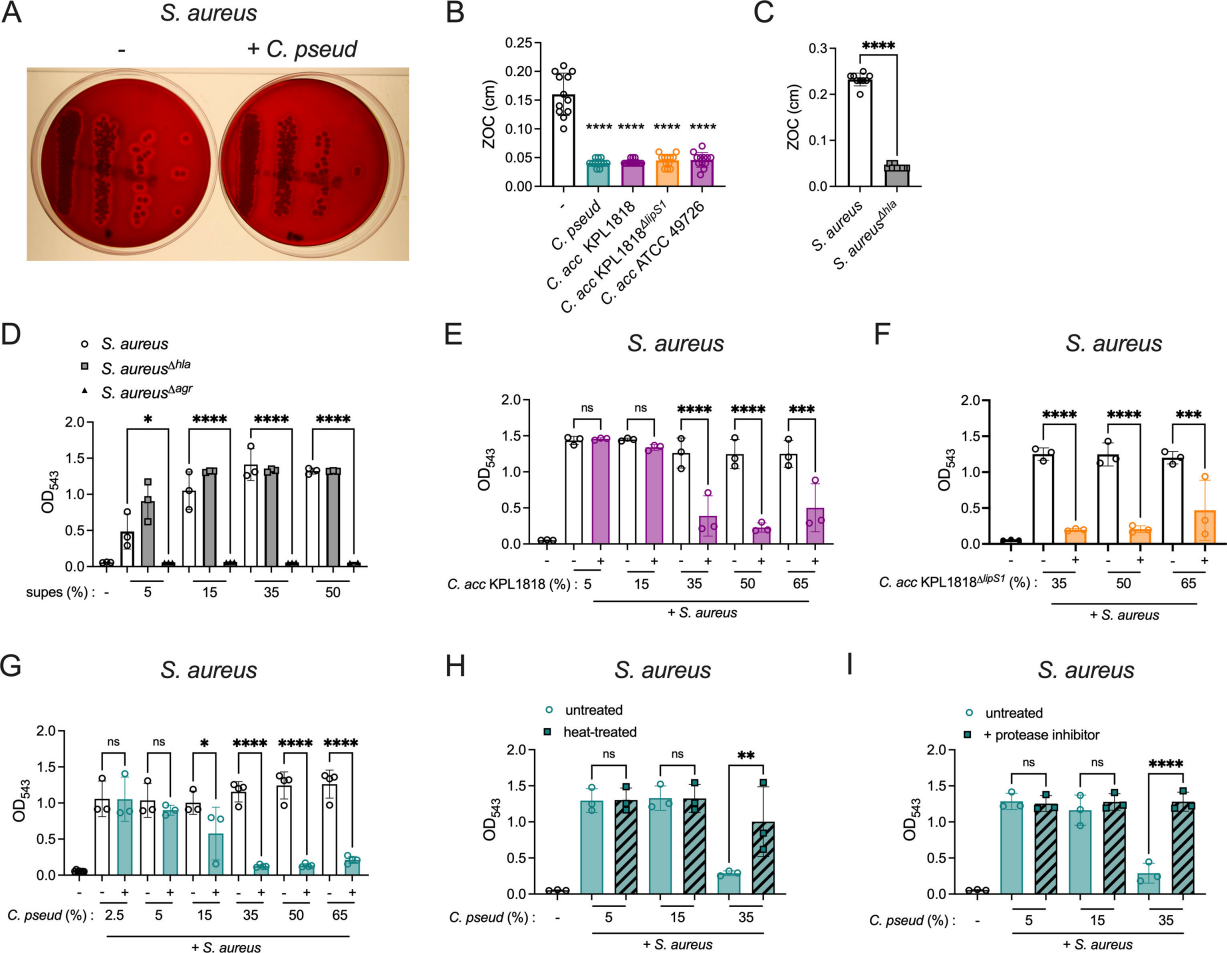
*Corynebacterium*-secreted factor directly inhibits *S. aureus* hemolysin activity. (**A**) *S. aureus* colonies on sheep blood agar plates with or without pre-spreading plates with filtered supernatants (CFCM) from *C. pseudodiphtheriticum* (*C. pseud*), with hemolysis visualized as cleared zones surrounding colonies. (**B**) ZOC quantified for *S. aureus* colonies on sheep blood agar plates with or without pre-spreading of CFCM from *C. pseudodiphtheriticum, C. accolens* WT (*C. acc* KPL1818), *lipS1*-deficient *C. accolens* (*C. acc* KPL1818^Δ^*^lipS1^*), or *C. accolens* ATCC 49726. (**C**) ZOC quantified for WT *S. aureus* compared with *hla*-deficient *S. aureus* (*S. aureus*^Δ^*^hla^*) on blood agar plates. (**D**) Hemolysis detected as OD_543_ of human red blood cells combined with the indicated percentage of filtered *S. aureus* supernatants from WT *S. aureus*, *S. aureus*^Δ^*^hla^*, and *S. aureus*^Δ^*^agr^*. (**E and F**) Hemolysis of human red blood cells combined with filtered *S. aureus* supernatant and the indicated percentage of filtered supernatants (CFCM) from *C. accolens* WT (*C. acc* KPL1818) (**E**), or *lipS1*-deficient *C. accolens* (*C. acc* KPL1818^Δ^*^lipS1^*) (**F**) or an equivalent percentage of growth medium alone. (**G**) Hemolysis of human red blood cells combined with filtered *S. aureus* supernatant and the indicated percentage of CFCM from *C. pseudodiphtheriticum* or an equivalent percentage of growth medium alone. (**H**) Hemolysis of human red blood cells as in (**G**) for *S. aureus* supernatants combined with untreated or heat-treated CFCM from *C. pseudodiphtheriticum*. (**I**) Hemolysis of human red blood cells as in (**G**) for *S. aureus* supernatants combined with CFCM from *C. pseudodiphtheriticum* with or without a protease inhibitor cocktail. *P < 0.05, **P < 0.01, ***P < 0.001, ****P < 0.0001, one-way ANOVA with Dunnett’s *post hoc* analysis (**B**), Tukey’s *post hoc* analysis (**D**), or Sidak’s *post hoc* analysis (**E–I**), or unpaired *t*-test (**C**). Data are representative of three experiments (**A**), pooled from three independent experiments with three replicates per group (**B**) or pooled from three to four independent experiments with one replicate per group (**E–I**).

While lipases secreted by *Corynebacterium* had no effect on *S. aureus* growth, we considered the potential effect of lipases or other secreted factors on *S. aureus* virulence. The *agr* quorum sensing system impacts the regulation of many key virulence factors in *S. aureus* (42, 43). Among these are the pore-forming toxins (PFTs), which lyse diverse host cell types including erythrocytes (44). CFCM from several *Corynebacterium* species were tested for an impact on *S. aureus* hemolysis using two different *in vitro* assays. First, *Corynebacterium* CFCM was spread onto 5% sheep blood agar plates before plating *S. aureus*. Hemolysis is visualized by the “aura” of lysed erythrocytes, quantified by ZOC. We found that several strains of *Corynebacterium* CFCM drastically reduced the hemolytic activity of *S. aureus*, visualized by a reduced transparent aura of lysed erythrocytes on the plates (Fig. 6A; Fig. S2A), and by the measured ZOC (Fig. 6B). This effect was lipase-independent, as both *C. accolens* and *C. accolens^Δlips1^* significantly reduced hemolytic activity. Sheep blood agar hemolysis was dependent on the PFT α-hemolysin (Hla), as hemolysis was absent in a *S. aureus^Δhla^* mutant (Fig. 6C). These data indicate that *Corynebacterium*-secreted factors inhibit α-hemolysin-dependent hemolysis. In contrast, we detected no effect for *Corynebacterium* CFCM on the characteristic “green halo” produced by *S. pneumoniae* in 5% sheep blood agar plates (Fig. S2B), indicating a selective effect on *S. aureus* hemolysis.

We next investigated *S. aureus* hemolysis of human blood using a spectroscopy assay. For this assay, *S. aureus* cultures were used to prepare *S. aureus* CFCM, which contains several PFTs, and hemolysis was measured following incubation with human red blood cells. Higher values of OD_543_ indicate greater levels of hemolysis, as reaction mixtures were centrifuged to pellet intact red blood cells. Importantly, human erythrocytes lack the receptor for α-hemolysin, ADAM10, rendering them insensitive to α-hemolysin-dependent hemolysis (45, 46). Consistent with this, CFCM from wild-type *S. aureus* and *S. aureus^Δhla^* resulted in similar levels of hemolysis (Fig. 6D). Human erythrocytes are sensitive to other *S. aureus* PFTs including leukocidins and phenol-soluble modulins (PSMs) (47, 48). *Agr* is critical for expression of both α-hemolysin and other PFTs capable of lysing human red blood cells (42, 47). In contrast to CFCM from wild-type *S. aureus*, CFCM from *agr*-deficient *S. aureus* exhibited no hemolytic activity (Fig. 6D), as expected. Thus, hemolytic activity measured in this assay with human red blood cells represents α-hemolysin-independent hemolysis. To assess the effect of *Corynebacterium* CFCM on *S. aureus* hemolysis of human red blood cells, *Corynebacterium* CFCM was combined with *S. aureus* CFCM and purified human erythrocytes. Both *C. pseudodiphtheriticum* and *C. accolens* CFCM significantly reduced *S. aureus* hemolysis, compared to control reactions without *Corynebacterium* CFCM (Fig. 6E through G; Fig. S2C). *C. accolens* CFCM inhibition did not require lipase activity, as *S. aureus* hemolysis was blocked by CFCM from both *C. accolens* and *C. accolens^Δlips1^* (Fig. 6E and F). *Corynebacterium* CFCM inhibition of *S. aureus* hemolysis was dose-dependent, with optimal inhibition above 15% CFCM (Fig. 6E and G). These data indicate that a secreted factor from *Corynebacterium* directly inhibits the activity of *S. aureus* hemolytic toxins.

To characterize the secreted factor(s) responsible, we compared activity of *C. pseudodiphtheriticum* CFCM after heat treatment and following incubation with a protease inhibitor cocktail. The inhibitor cocktail consisted of AEBSF, Aprotinin, Bestatin, E64, Leupeptin, and Pepstatin A inhibitors, providing broad-spectrum inhibition of activity by serine proteases, cysteine proteases, aspartic acid proteases, aminopeptidases, and metalloproteases. Heat-treated *Corynebacterium* CFCM displayed a significantly reduced effect, as *S. aureus* CFCM hemolysis was largely restored (Fig. 6H), suggesting that the secreted factor is heat-labile. Incubation of *C. pseudodiphtheriticum* CFCM with a protease inhibitor cocktail also restored *S. aureus* hemolysis activity (Fig. 6I). Control reactions with DMSO alone had no effect on *S. aureus* hemolysis (Fig. S2D). Finally, *C. pseudodiphtheriticum* CFCM activity was lost after passage through a 10 kD molecular weight filter, but retained in the >10 kD fraction, suggesting that the hemolysis inhibitory factor is not a small molecule or metabolite (Fig. S2E). Together, these findings identify a *Corynebacterium*-secreted factor whose protease activity directly inhibits *S. aureus* hemolysis.

## DISCUSSION

The airway epithelium is a battleground for the millions of bacteria colonizing our respiratory tract, primarily residing in the upper airway. Classified by the World Health Organization as “priority pathogens,” *S. aureus* and *S. pneumoniae* frequently colonize the upper airway and cause bacterial pneumonia upon invasion to the lungs. In contrast, *Corynebacterium* function largely as commensal bacteria in the airway and correlate with reduced respiratory tract infection risk (17–20). This study expands our understanding of the mechanisms by which *Corynebacterium* impact *Staphylococcus aureus* and *Streptococcus pneumoniae* pathogenesis by defining species-conserved and species-specific effects against pathogen infection, adherence to the airway epithelium, growth, and virulence.

Adherence to the respiratory tract epithelium is a key pre-requisite for colonization, infection, and future disease spread for both *S. pneumoniae* and *S. aureus*. The polysaccharide capsule of *S. pneumoniae* plays an important role in defense against host immune responses (49). However, during colonization, *S. pneumoniae* sheds its capsular polysaccharide to promote epithelial adherence through increased exposure of cell adhesion molecules (50), motivating our use of unencapsulated R6 to assess pneumococcal adherence to respiratory tract epithelial cells. *S. aureus* adherence is largely regulated by microbial surface components recognizing adhesive matrix molecules (MSCRAMMs) (51). The fibronectin-binding MSCRAMMs FnbA and FnbB are critical for colonization and invasion of host tissue (39, 52, 53). Here, we found that *Corynebacterium* successfully colonize D562 and A549 respiratory tract epithelial cells, providing a new investigative tool to dissect *Corynebacterium* interactions with host cells and other bacteria. These studies identify two *Corynebacterium* species, *C. pseudodiphtheriticum* and *C. accolens*, which broadly inhibit adherence of *S. pneumoniae* and *S. aureus* to human respiratory tract epithelial cells. Additional studies will be necessary to determine whether other *S. pneumoniae* and *S. aureus* isolates are similarly affected. The mechanism for *C. pseudodiphtheriticum* interference with pathogen adhesion is likely related to direct competition from the colonizing *Corynebacterium*, suggested by the dose dependence of this effect, requirement for live *Corynebacterium*, and limited effect on *S. aureus* mutants with significantly low or high baseline adherence. This aligns well with the current model for pathogen interference from resident microbial populations colonizing the mucosal surface, which limit the available “real estate” on the epithelium for pathogens to establish a niche. While *C. accolens* inhibition of *S. aureus* adherence also required live bacteria, this strain was still effective against highly adherent and low adherent *S. aureus*, indicating strain-dependent differences for this phenotype and the potential involvement of factors beyond epithelial colonization, as both strains colonized similarly well. Beyond direct niche competition, secreted proteases from *Corynebacterium*, such as those responsible for directly impairing *S. aureus* hemolysis, may alter adherence factors including FnbA and FnbB or interfere with their binding. The effect of *Corynebacterium* protease activity on *S. aureus* and *S. pneumoniae* adherence and the mechanisms underlying strain-dependent differences remain important areas for further investigation.

In mice, we found that pre-exposure to *C. pseudodiphtheriticum* reduced colonization and lung infection with both *S. pneumoniae* and *S. aureus*, though the effect was more pronounced against *S. pneumoniae*. In prior work, we established that *C. accolens* similarly reduced *S. pneumoniae* infection, and this effect was partially dependent on LipS1 (23). Secreted lipases including *C. accolens* LipS1 directly inhibit pneumococcal growth through the release of bactericidal free fatty acids following host lipid catabolism (32). *C. accolens* is lipophilic, as it requires free fatty acids to grow (54). However, several non-lipophilic *Corynebacterium* species including *C. pseudodiphtheriticum*, *C. striatum*, and *C. amycolatum* demonstrated lipase activity, as *S. pneumoniae* growth was only inhibited in the presence of the host lipid triolein. Among these, we previously identified a lipase in *C. amycolatum* responsible for triolein-dependent inhibition of *S. pneumoniae* (23). In *C. pseudodiphtheriticum*, the protein with highest homology to LipS1 is WP_244266826.1, categorized as an alpha/beta hydrolase family protein, like LipS1, in the NCBI Conserved Domain Database (55). This protein, potentially together with other lipases expressed by *C. pseudodiphtheriticum*, may contribute to triolein hydrolysis in this strain. In addition to triolein digestion by LipS1, bacterial lipases and esterases can hydrolyze Tween 80, used in the growth medium, serving as an additional source of free fatty acid release as we observed for the *Corynebacterium* strains that inhibited *S. pneumoniae* without triolein. Regardless, lipase expression did not contribute to reduced pneumococcal adherence to respiratory tract epithelial cells colonized with *C. accolens*. The concentration of *Corynebacterium*-secreted lipase, along with the proximity or availability of host-derived lipid, was likely insufficient to mediate direct growth inhibition in this setting. Future work in air liquid interface (ALI) cultures, where cells are not submerged in culture media, may better facilitate investigation of lipase-dependent effects. The use of more complex epithelial models, such as primary human epithelial ALI cultures comprised of ciliated and mucous secreting cells, is an important next step in translating the findings presented here to understand *Corynebacterium* dynamics with bacterial pathogens occurring at the airway epithelium. Regardless, these findings suggest that both lipase-dependent and -independent mechanisms contribute to *Corynebacterium*-mediated protection against pneumococcal colonization and infection.

The *agr* quorum sensing system in *S. aureus* regulates secretion of a large suite of virulence factors and adhesion molecules. In response to higher cell population density and increased levels of autoinducing peptide (AIP), *agr* activation leads to increased expression of virulence factors while adhesion is downregulated (40, 56–58). This process reflects the stages of *S. aureus* infection; upon initial tissue colonization, adhesion factors are upregulated, but as pathogen cell density increases, the population requires more nutritional resources and immune evasion mechanisms become critical (59–61). *Agr* is required for *S. aureus* invasion of the lungs and increases *S. aureus*-associated mortality (62, 63). *Agr* has also been implicated in *Corynebacterium-Staphylococcus* interactions, as *S. aureus* with spontaneous *agr* mutations survive contact-dependent growth inhibition by *C. pseudodiphtheriticum* on agar plates (64). Prior work by Ramsey et al. found that CFCM from *Corynebacterium striatum* reduced *S. aureus agr* activation (65). In their study, *S. aureus* incubated with *C. striatum* CFCM in the presence of AIP was more adherent to A549 epithelial cells. These data align with our finding that *S. aureus* adherence is increased in the absence of *agr*. Increased adherence in *agr*-deficient *S. aureus* may result from relief of fibronectin-binding protein inhibition and reduced production of protease V8, which cleaves fibronectin-binding proteins (66). However, live colonizing *Corynebacterium* had the opposite effect, and instead reduced *S. aureus* adherence to epithelial cells. These findings suggest that *Corynebacterium* can interfere with initial *S. aureus* attachment to the respiratory epithelium. However, once *S. aureus* is established and AIP-induced *agr* is triggered, *Corynebacterium*-secreted factor(s) reported in Ramsey et al. may reduce virulence. It remains unclear whether these mechanisms occur simultaneously or compete with one another during *S. aureus* colonization and infection *in vivo*.

*S. aureus* PFTs, which are regulated by the *agr* system, can lyse an array of human cell types, including monocytes, neutrophils, dendritic cells, lymphocytes, epithelial cells, and erythrocytes (44, 67). The major *S. aureus* PFT categories are hemolysins (α-, β-, and γ-), leukocidins, and PSMs. These toxins are critical for virulence, playing an important role during *S. aureus* pneumonia (63), skin infection (68), and sepsis (69). In the previously mentioned study by Ramsey et al., *S. aureus* hemolysis was reduced following growth in *C. striatum* CFCM, which downregulated *S. aureus agr* activity and virulence gene expression in the presence of AIP. Unexpectedly, we found that CFCM from multiple *Corynebacterium* strains directly interfered with the activity of *S. aureus* hemolysins, as *Corynebacterium* CFCM was added to *S. aureus* CFCM, rather than live cells, without the opportunity to affect gene expression. In this setting, *Corynebacterium* CFCM was sufficient to block both α-hemolysin-dependent and -independent hemolysis, indicating interference with multiple PFTs. This anti-hemolytic activity was abolished in the presence of a protease inhibitor, suggesting that a *Corynebacterium*-secreted protease is responsible. In contrast to this unidentified protease, which we found was larger than ~10 kD, the secreted factor described by Ramsey et al. to alter *S. aureus* gene regulation was a small molecule under 3 kD. Together, these findings indicate that distinct *Corynebacterium*-secreted factors modulate *S. aureus* virulence gene expression and directly interfere with PFT activity. *S. pneumoniae* growth on blood agar plates produces a “green halo” often described as α-hemolysis, due to hydrogen peroxide oxidation of oxy-hemoglobin (70). *Corynebacterium*-secreted factors had no impact on *S. pneumoniae* hemoglobin oxidation, which occurs through alternative mechanisms to *S. aureus* PFTs.

The commensal *Staphylococcus epidermidis* also secretes a factor in CFCM that impairs *S. aureus* hemolysis, though this effect was mediated by regulation of *S. aureus* gene expression (71), as reported for *C. striatum. S. aureus* produces several proteases including aureolysin, a metalloproteinase that modulates hypervirulence in *S. aureus* infection by preventing accumulation of α-hemolysin and phenol-soluble modulins (72–74). While we were unable to identify aureolysin homologs in the *Corynebacterium* strains used in this study, the *Corynebacterium*-derived protease(s) we describe may interfere with *S. aureus* hemolysis through a similar mechanism. The *Corynebacterium* strains investigated in this study encode genes for several classes of proteases including metalloproteases, serine proteases, and cysteine proteases. These include proteases with homologs in the exoproteome of *Corynebacterium diphtheriae*, indicating they are likely secreted (75). *Corynebacterium* proteases could also influence other *S. aureus* phenotypes including adherence, as mentioned above, if fibronectin-binding proteins are targeted similar to *S. aureus*-derived V8 protease (66). It was recently shown that PSMs secreted by *S. aureus* can interfere with *C. pseudodiphtheriticum* aggregation and colonization of nasal epithelial cells (76), suggesting bi-directional interactions between *Corynebacterium* and *S. aureus* PFTs.

Overall, these findings support the notion that commensal *Corynebacterium* species in the upper airway contribute to the beneficial effect of the respiratory tract microbiome by identifying new mechanisms by which *Corynebacterium* species protect against colonization and lung infection by opportunistic pathogens. These protective mechanisms include interference with pathogen adherence to the respiratory tract epithelium as well as the secretion of factors that impede pneumococcal growth and *S. aureus* virulence. Identifying the conditions under which *Corynebacterium* impairs pathogen colonization and the secreted factors regulating their growth and virulence is a critical next step toward developing *Corynebacterium*-based therapeutics.

## AFTER EPUB

*[This article was published on 20 December 2024 with an error in the abstract. The first line of the abstract was corrected in the current version, posted on 8 January 2025.]*

## SUPPLEMENTAL MATERIAL

Supplemental material iai.00445-24-s0001.pdf Fig. S1 and S2.

ASM does not own the copyrights to Supplemental Material that may be linked to, or accessed through, an article. The authors have granted ASM a non-exclusive, world-wide license to publish the Supplemental Material files. Please contact the corresponding author directly for reuse.
