# Supplementary material for: Airway Corynebacterium interfere with Streptococcus pneumoniae and Staphylococcus aureus infection and express secreted factors selectively targeting each pathogen
Source: Infect Immun. 2024 Dec 20;93(2):e00445-24. doi: 10.1128/iai.00445-24 (PMC11834435; doi:10.1128/iai.00445-24)
Supplement: Supplemental material — Fig. S1 and S2. [file iai.00445-24-s0001.pdf]

SUPPORTING INFORMATION

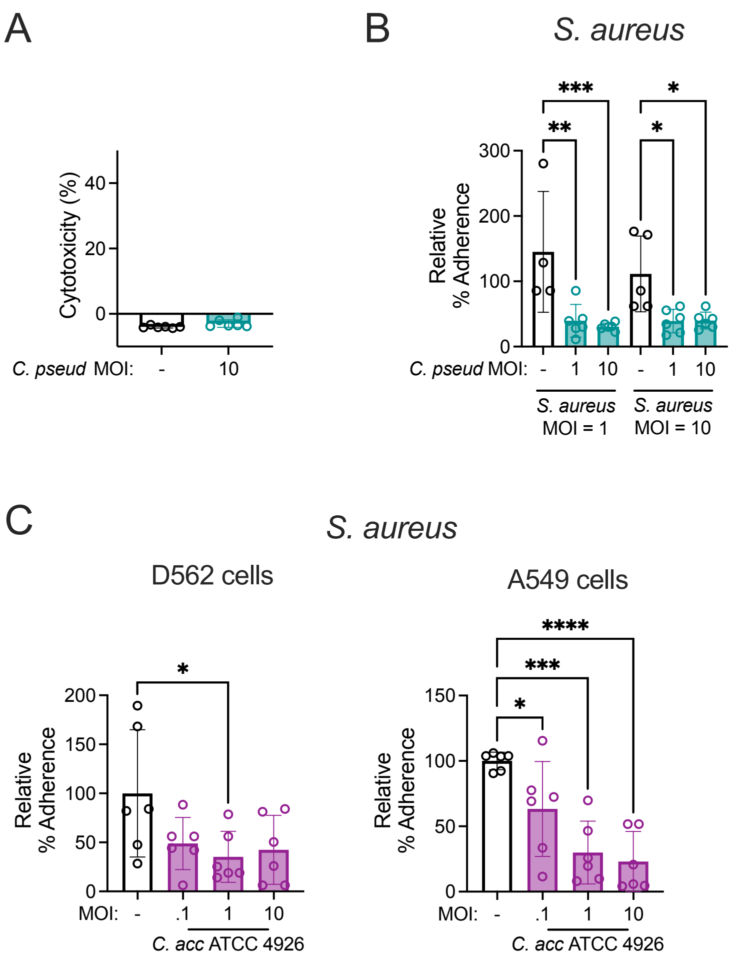

Supplemental Figure 1. *S. aureus* adherence is reduced by *C. pseudodiphtheriticum* colonization when infected with a higher MOI and following colonization with *C. accolens* strain 4926. (A) Percent cytotoxicity detected by measurement of lactate dehydrogenase (LDH) release in the supernatant of A549 cultures colonized with or without *C. pseudodiphtheriticum* for 18 hours at the indicated MOI. (B) Percent adherence of *S. aureus* MRSA strain USA300 detected on A549 cells at 1 hour post-infection with *S. aureus* MOI 1 or MOI 10, with or without pre-colonization of epithelial cells with *C. pseudodiphtheriticum* for 18 hours at the indicated MOI, relative to untreated cells infected with *S. aureus* alone. (C) Percent adherence of *S. aureus* detected on D562 or A549 cells at 1 hour post-infection with or without pre-colonization of epithelial cells with *C. accolens* strain ATCC 4926 (*C. acc* ATCC 4926), for 18 hours at the indicated MOI, relative to untreated cells infected with *S. aureus* alone. **p*<.05, ****p*<.001, *****p*<.0001, one-way ANOVA with Dunnett's post-hoc analysis. Data are pooled from two independent experiments with three replicates per condition.

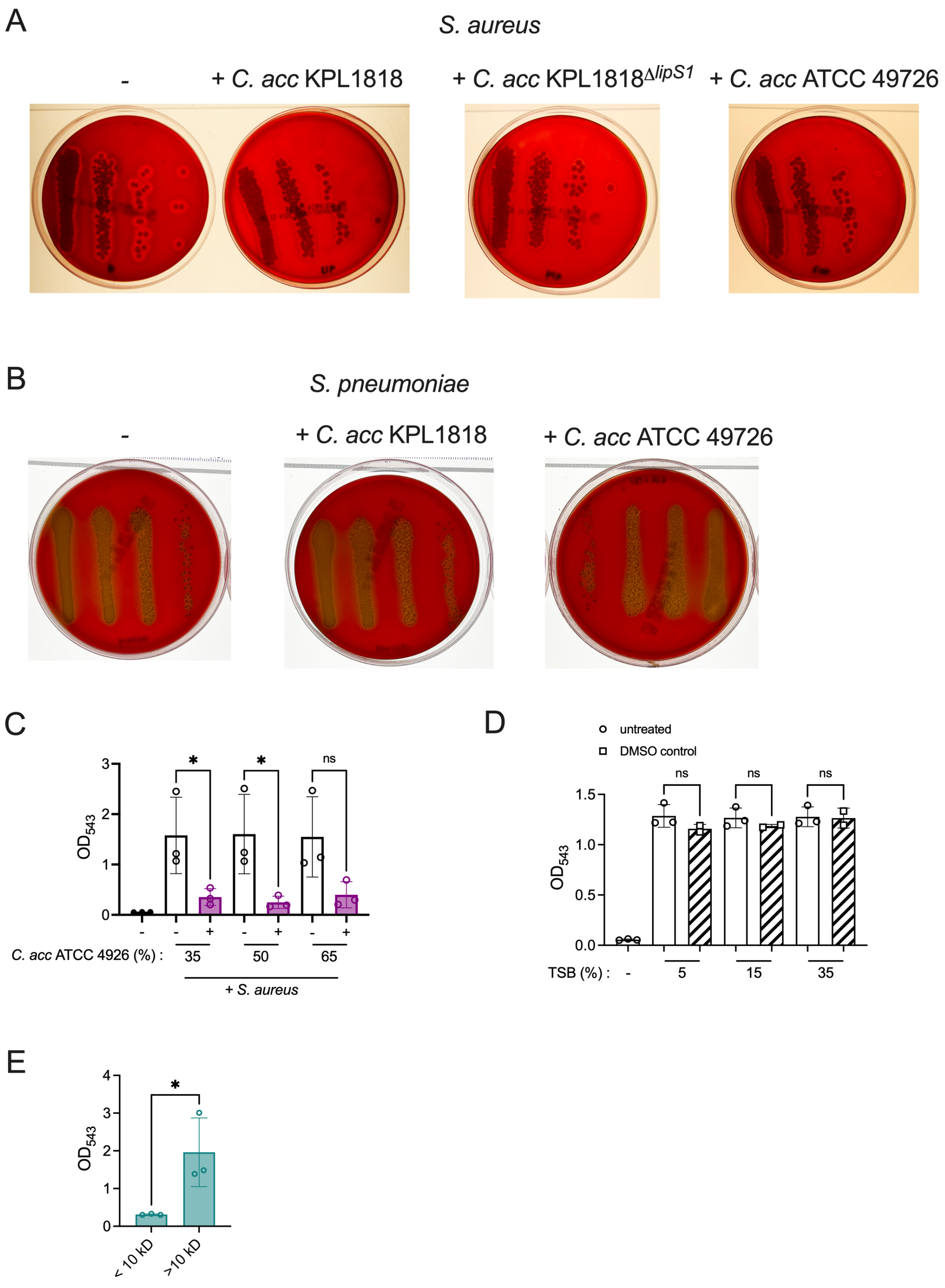

Supplemental Figure 2. *Corynebacterium* secreted factor >10 kD directly inhibits *S. aureus* hemolysis. (A) *S. aureus* colonies on sheep blood agar plates with or without pre-spreading plates with filtered supernatants (CFCM) from *C. accolens* (*C. acc* KPL1818), *lipS1* deficient *C. accolens* (*C. acc* KPL1818$^{\Delta lipS1}$), or *C. accolens* strain ATCC 4926 (*C. acc* ATCC 4926), with hemolysis visualized as cleared zones surrounding colonies. (B) *S. pneumoniae* colonies on sheep blood agar plates with or without pre-spreading plates with CFCM from *C. accolens* KPL181 or *C. accolens* ATCC 4926. (C) Hemolysis of human red blood cells combined

with filtered *S. aureus* supernatant and the indicated percentage of CFCM from *C. accolens* ATCC 4926 or an equivalent percentage of growth medium alone. (D) Hemolysis of human red blood cells for *S. aureus* supernatants combined with growth medium with or without DMSO. (E) Hemolysis of human red blood cells for *S. aureus* supernatants combined with CFCM from *C. pseudodiphtheriticum* that either passed through a 10 kD filter (<10 kD) or were retained (>10 kD). **p*<.05, one-way ANOVA with Sidak's post-hoc analysis (C-D) or unpaired t test (E). Data are representative of three independent experiments (A-B) or pooled from three independent experiments (C-E).
